# Evaluation of Shear and Peel Strength of Al1060 Single-Lap and T-Lap Joints Produced by Rotated Clinching Process with Twin Rotating Punches

**DOI:** 10.3390/ma15124237

**Published:** 2022-06-15

**Authors:** Yulin He, Lianfa Yang, Jing Dang, Aliang Gao, Wenze Zhang

**Affiliations:** School of Mechanical and Electrical Engineering, Guilin University of Electronic Technology, Guilin 541004, China; hyl_2005@126.com (Y.H.); 18837188389@163.com (J.D.); aliang_gao@163.com (A.G.); 15234186554@163.com (W.Z.)

**Keywords:** rotated clinching process, strength, mechanical loads, cross-sectional profile, failure mode

## Abstract

The clinching process is widely used in joining lightweight sheet metal. We proposed a novel rotated clinching process (RCP), which is characterized by a flat bottom die structure and twin rotating punches. The aim of this study was to evaluate the strength of RCP joints. Al1060 sheets with thicknesses of 1.5 mm and 2 mm were used as the experimental materials. Overlap and T-lap RCP joints with three die depths and five bottom thicknesses were fabricated, and shear and peel tests were performed on the joints. The joint strengths were evaluated based on the mechanical load, cross-sectional profile dimensions, and failure mode. The results showed that the mechanical load is a direct, reliable, and quantitative evaluation criterion, while the cross-sectional profile and failure mode are indirect and qualitative. These criteria confirmed that the strength of thick sheet joints is higher than that of thin sheet joints, the shear strength is superior to the peel strength, and the strengths of the joints are high with failure mainly occurring due to tearing or shear failure. Finally, the key parameters for determining the strength of a joint include the bottom thickness/sheet thickness ratio (*R*_t_), and the die depth (*h*).

## 1. Introduction

To save energy and reduce pollution, lightweight materials such as aluminum alloys and high-strength steels are commonly used in the manufacturing industry. These materials are connected together by several different methods. Owing to their different properties, joining different materials using the popular resistance spot welding method can be difficult. Friction stir welding has several advantages over conventional welding methods and is widely used in light metal structures such as aluminum alloys and magnesium alloys. Moradi et al. [1] investigated the effects of welding variables on the joint using a finite element method; however, the joint was softened because of the effects of thermal cycling that caused the mechanical properties of the joint to deteriorate. For the welding of heat-sensitive alloys, Vahdati et al. [2] researched a new submerged friction stir welding method. Although this method reduced the thermal effect by using cooling fluid, the mechanical properties of the joint were affected by the microstructural transformation of the material due to severe plastic deformation. Therefore, people resort to other mechanical connection methods, such as self-piercing riveting and clinching. In the self-piercing riveting method, the sheets are joined by piercing the upper sheet with rivets. The cost and weight of the joint are higher due to the additional rivets, and the corrosion resistance of the joint may be insufficient. In contrast, clinching uses a special punch and die to join sheets together by plastic deformation, so no additional fasteners are required, and the joint is lightweight. The clinching technique has attracted increasing attention in the manufacturing industry, and new clinching methods are being developed.

One such new method is the rotated clinching process (RCP), which we developed [3]. Different from traditional clinching, the RCP uses twin rotating punches instead of a single linear punch, and a flat bottom die instead of a grooved die. During forming, the twin punches rotate at a constant speed to punch and plastically deform the sheets into the die cavity. The result of this is a joint with two cavities on the twin punches side and a flat bottom on the die side, which cannot be easily rotated under torque. The RCP can be used to continuously join sheets at multiple points without damaging their surfaces.

However, the strength of the joint is an important characteristic that needs to be evaluated. Some researchers have determined the strength of the clinched joint using mechanical tests for different cross-sectional profile dimensions and failure modes. Mechanical tests such as shear, tensile, peeling, and fatigue tests are performed on the clinched joint, and the strength of the joint (including static strength and fatigue strength) can be evaluated directly from the mechanical loads. Carboni et al. [4] and Abe et al. [5] performed mechanical tests on the joints of several different sheets and found that the fatigue strength was higher than the static strength of the joints. Mori et al. [6] and Su et al. [7] performed mechanical tests on self-piercing riveting, resistance spot welding, and clinched joints and found that the fatigue and static strength of the self-piercing riveting joints were the best. The static strength of the clinched joints was the worst, and the fatigue strength of the clinched joints was equal to that of resistance spot welding. It can be seen, therefore, that the clinched joints have good fatigue strength, but poor static strength, so researchers are paying more attention to the evaluation of the static strength of the clinched joint, such as the shear, peel, and tensile strength. These strengths depend on the cross-sectional profile of the joint (neck thickness, interlock value, and bottom thickness), and are different for different joint failure modes. A high neck thickness and interlock value generate high strength joints. Therefore, in addition to the mechanical tests, the strength of the joint can also be evaluated by cross-sectional profiles and failure modes. Lambiase [8] measured the cross-sectional profile dimensions of the joints of different die structures and performed cross-tensile mechanical tests. It was found that the neck thickness and interlock value can increase with appropriate die structure parameters, improving the tensile strength. Wen et al. [9] measured the cross-sectional profile dimensions of compression joints and tested their shear mechanics. The results showed that the protruding height could be reduced, and the neck thickness and interlock value could be increased by using a pair of flat bottom dies to reshape the clinched joints. In addition, joints reshaped by a flat die and a bump die exhibited a better neck thickness and higher shear strength [10]. Li et al. [11] measured the cross-sectional profile dimensions and tested the shear mechanics of a steel aluminum alloy clinched joint. When the ratio of neck thickness to interlock value was less than or close to 1, the shear strength increased by 16.94%. Jiang et al. [12] performed the same study on the joint of galvanized SAE1004 steel and pre-strained aluminum AA6111-T4, and the shear strength was high when the bottom thickness was small. He et al. [13] performed shear mechanical tests and a failure mode analysis on clinching-bonded hybrid joints and found that the shear strength of the hybrid joints with neck fracture failure was higher than that of clinched joints. Ran at al. [14] performed mechanical shear and tensile tests on rectangular joints and analyzed the failure modes. They found that the shear strength and tensile strength were higher when the neck fracture occurred. Peng et al. [15] conducted shear mechanical tests, cross-sectional profile measurements and failure mode analysis on two-stroke flat clinched (TFC) joints and clinched joints of Al1060 aluminum sheets. It was found that the neck thickness, interlock value, and shear strength of TFC joints are higher than those of clinched joints, and the failure modes are neck fractures. Lee et al. [16] also performed the same investigation on the hole-clinched joints of carbon fiber reinforced plastic (CFRP). It was found that the thicker the CFRP and the higher interfacial strength at the CFRP interface led to greater shear strength. In addition, the joint with a larger hole diameter in the CFRP had a high shear strength, and the failure mode was determined by the ply angle of CFRP sheets [17]. Chen et al. [18] performed mechanical shear and tensile tests on the joints of different sheets using flat bottom clinching and analyzed the cross-sectional profile and failure modes. It was found that the joint with a hard upper sheet exhibited a larger neck thickness and a lower interlock value, as well as larger joint strength. Neck fracture failure occurred in the shear test and unbuttoning failure occurred in the tensile test. In addition, the strengths can be substantially improved by adding rivets at the joints [19]. Babalo et al. [20] also evaluated the electrohydraulic clinching joint. The results showed that the tensile strength was higher than the shear strength when neck fracture failure occurred at a large interlock value. Zhao et al. [21] also evaluated the round joint and rectangular joint, and the results showed that the rectangular joint had a small bottom thickness and a large interlock value, and the shear strength was 1.7 times that of the round joint when mixed failure of neck fracture and unbuttoning occurred.

These results show that the strength of the joint is influenced by the neck thickness, interlock value of the cross-section, and the failure mode. At present, there is little in the literature about the shear and peel strength of RCP joints. In this study, the experimental method was designed to evaluate the joint strengths based on mechanical tests, cross-sectional profile dimensions, and failure modes. For this purpose, Al1060 sheets with distinct thicknesses were first joined by RCP at three die depths and five bottom thicknesses, and joints with different parameters were fabricated. Mechanical shear and peel tests were then performed on these joints, their cross-sectional profile dimensions were measured, and the failure modes of joints were observed and analyzed. Finally, a parameter range for better shear and peel strength and the parameters for the best shear and peel strength were determined. The research results can serve as a reference for the practical application of RCP. The principles of the RCP are described in Section 2. Section 3 describes the joining experiments that were conducted using RCP, the shear and peel tests, the measurements of cross-sectional profile dimensions, and the failure mode analyses. Subsequently, the shear and peel loads, the cross-sectional profiles in terms of interlock and neck thickness, and the failure modes of RCP joints were analyzed in Section 4, which led to the conclusions presented in Section 5.

## 2. Forming Principle of RCP

**Figure 1:**
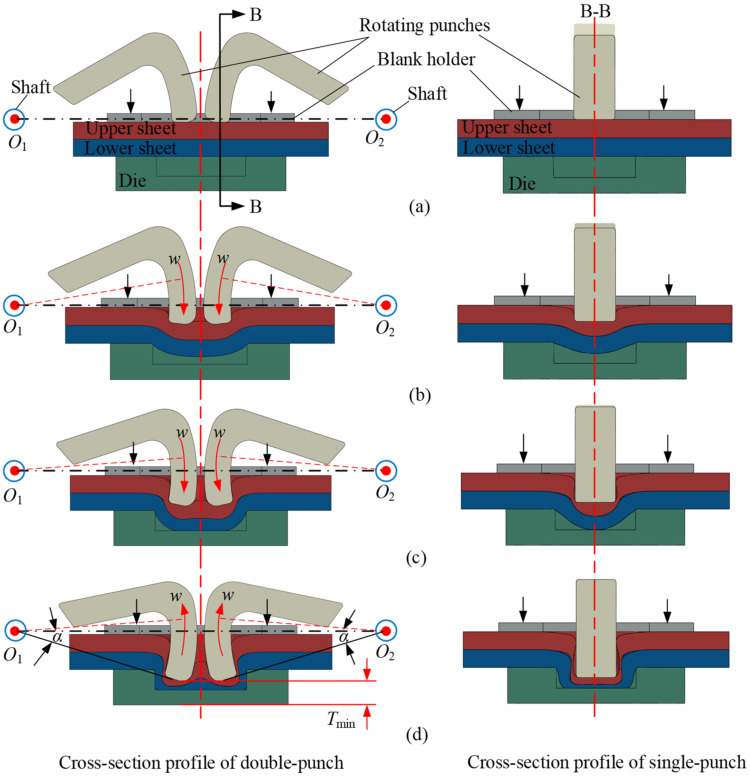
Forming processes of the RCP joint. (**a**) Positioning, (**b**) extrusion, (**c**) filling and (**d**) forming.

The RCP is a cold forming technology in which two sheets are subjected to extrusion through twin rotating punches and a flat bottom die. The material flows laterally in the die cavity, resulting in interlocking between sheets, forming an uncircular joint. The joint forming process goes through the following four stages, which are shown in Figure 1. (a) Positioning: the upper and lower sheets are placed on the die and clamped with a blank holder to prevent warping. Twin rotating punches are each installed on the shaft and aligned with the die cavity. (b) Extrusion: the twin rotating punches rotate around the centers *O*_1_ and *O*_2_ at a constant speed (*w*). The upper and lower sheets are extruded and flow into the die cavity, and a slight plastic deformation begins to occur. (c) Filling: the twin rotating punches continue to rotate, resulting in a severe plastic deformation of the two sheets and gradually filling them all around. (d) Forming: under the continuous rotation of the twin rotated punches, the upper and lower sheets continue to flow around the die cavity. When the setting rotation angle (α) is reached, the twin rotating punches rotate in the opposite direction to demold, and an interlocking is formed between the upper and lower sheets. A joint with two cavities on the side of the punches and a flat bottom on the side of die is obtained. Joints with different bottom thickness (*T*_min_) can be obtained by setting different rotation angles (*α*). In Figure 1, the cross-sectional profile of a double-punch is shown on the left and that of a single-punch on the right cutting along the B-B axis.

## 3. Experimental Procedures

To evaluate the strength of the RCP joints, it is important to obtain the shear and peel loads, cross-sectional profile dimensions, and failure modes of the joints. The purpose of this section is to describe the experiments and measurements performed on the joints. First, the RCP joining experiments were conducted with different parameters (two sheet thicknesses, three die depths, and five bottom thicknesses) to obtain the RCP joints. Mechanical shear and peel tests were then performed on these joints, and their cross-sectional profile dimensions and failure modes were measured and observed.

### 3.1. Joining Experiments

Al1060 aluminum with good ductility and corrosion resistance is widely used in industrial production, and therefore it was selected as the joining material for the RCP experiments. The thicknesses of the Al1060 aluminum sheets were 1.5 mm and 2 mm, the modulus of elasticity was 70.2 GPa, the tensile strength was 114.6 MPa, and the yield strength was 95.3 MPa. To ensure the consistency of material properties, all specimens were made from the same batch of metal sheet, which were cut into strips with a nominal size of 80 mm × 20 mm.

**Figure 2:**
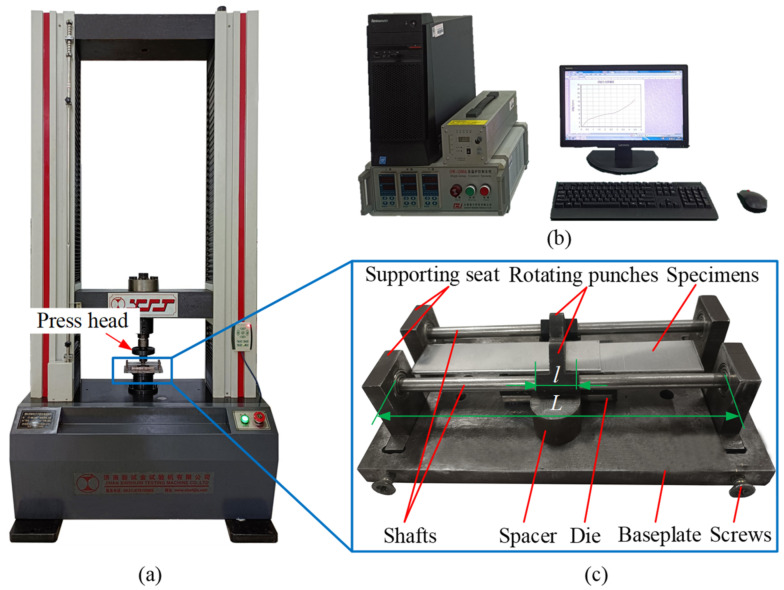
The RCP joining experimental platform. (**a**) Power system, (**b**) data acquisition system and (**c**) experimental apparatus.

**Figure 3:**
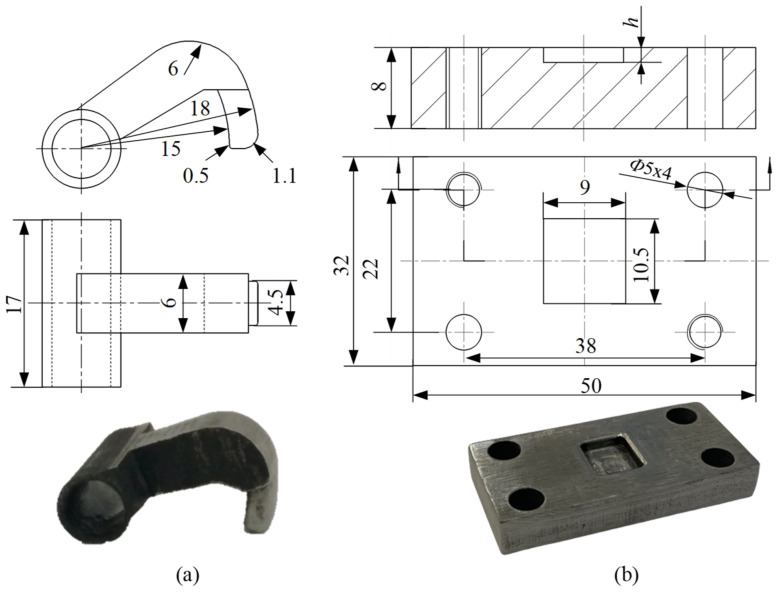
The structure and physical images of the (**a**) rotating punches and (**b**) stationary die (all dimensions in mm).

**Figure 4:**
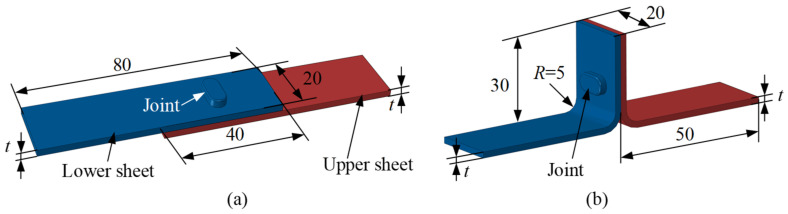
The structural dimensions of (**a**) single-lap and (**b**) T-lap samples (all dimensions in mm).

Figure 2 shows the RCP experimental platform, which consisted mainly of the power supply system, data acquisition system, and experimental equipment. The power supply system comprised a WDW-100GD (Jinan New Gold Testing Machine Co., LTD, Jinan, China) microcomputer-controlled testing machine (see Figure 2a) which provided the power for the joining experiment. The data acquisition system collected the load-stroke data during the forming process (see Figure 2b). The experimental apparatus was used to join specimens (see Figure 2c). It mainly comprised twin rotating punches, two shafts, and one die. The twin rotating punches were each fixed on one of two shafts and they rotated around the shafts. The shafts were installed in the four bearing seats, fixed to the base plate, and the center distance between the two shafts was adjusted by screws. Figure 3 shows the structural design and physical images of the twin rotating punches and the die, and the physical objects can be seen after heat treatment and preliminary grinding. To realize the movement of the twin rotating punches, according to the principle of gear meshing, the part assembled by the punches and the shafts was designed as a circular ring. The part of the rotating punches that impacts on the upper sheet was designed as an arc surface with an internal and external radius of 15 mm and 18 mm, respectively. This is conducive to forming an interlock, and to demolding for rotation in the reverse direction after forming. The top surface of the rotating punch was intentionally designed as an arcuate surface with a radius of 6 mm, so that it moved in a circular manner under the action of the press head. The bottom of the die was flat, which is simpler than the traditional grooved die. In this study, three values of die depth (*h*) were selected, of 2.2 mm, 2.3 mm, and 2.4 mm, respectively. To facilitate the installation of a single-lap sample (see Figure 4a), the length (*L*) of the shafts was approximately 8 times the length (*l*) of the rotating punches. To prevent the shaft from bending during the joining experiment, in addition to ensuring the rigidity and strength of the shaft, a spacer can be placed under the rotating punch, as shown in Figure 2c.

The RCP joining experiments were performed on two types of lap samples. The structural dimensions of the lap samples are shown in Figure 4, which consist of upper and lower sheets with an individual thickness (*t*) of 1.5 mm or 2 mm. Single-lap and T-lap samples were used to test the shear and peel strengths. The press head speed was set to 10 mm/min, the bottom thickness (*T*_min_) of the joint (as shown in Figure 1) was controlled by the stroke of the press head and the strokes were set to 0.7, 0.6, 0.5, 0.4 and 0.3 of the sheet thickness (*t*), respectively. During the joining experiments, the press head moved downward and acted on the top of the twin rotating punches which rotated around the shafts. When the bottom thickness reached the set value, the press head stopped its downward movement, and then moved upward. The rotating punches rotated backwards to perform demolding and a joint was made. To obtain different joints, RCP joining experiments were performed with five different bottom thicknesses for each die depth.

### 3.2. Shear and Peel Tests

**Figure 5:**
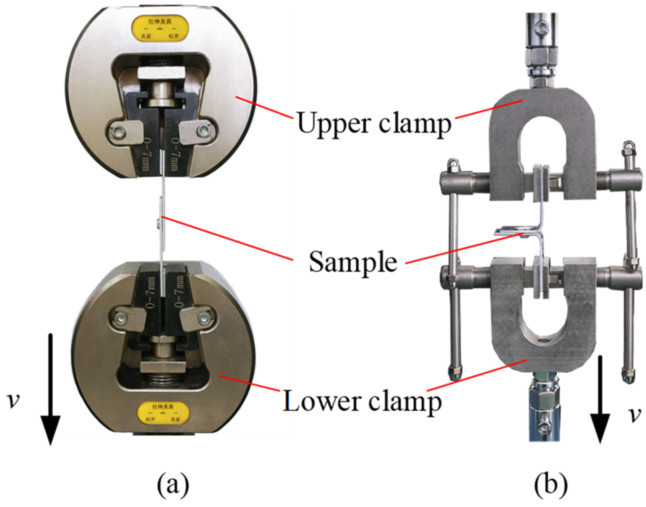
Tests of (**a**) shear and (**b**) peel.

Shear and peel tests can reflect the shear and peel resistance of the joint, and the maximum load obtained during the test can be used to evaluate the joint strength. To determine the maximum shear and peel load of RCP joints, shear and peel tests must be performed, which were conducted on the WDW-10 microcomputer-controlled testing machine. Figure 5 shows the clamping methods of the single-lap and T-lap samples. To prevent the torque from affecting the shear test results during clamping, a spacer of the same thickness as the sheet was placed at both ends of the sample. During the tests, the upper clamp was fixed and the movement speed (*v*) of the lower clamp was set to 2 mm/min [16]. When the two sheets were completely separated, the lower clamp stopped its movement, and the load–stroke curve with the stroke as abscissa and load as ordinate was obtained. The maximum load on the curve is considered to be the joint strength. To reduce the test error, the sample was tested three times for each parameter and the average value was obtained.

### 3.3. Dimensions of Cross-Sectional Profile

**Figure 6:**
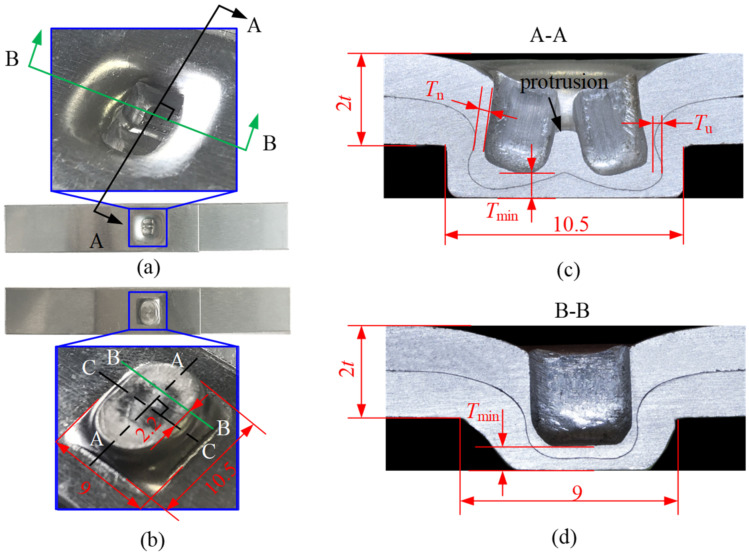
Images of the RCP joint (all dimensions in mm). (**a**) On the rotating punches side, (**b**) on the die side, (**c**) double- punch cross-section and (**d**) single-punch cross-section.

The cross-sectional profiles of the joint may directly reflect the interlock, neck thickness, and bottom thickness. The RCP joint is shown in Figure 6, where AA and CC lines are the symmetrical center of the joint. From Figure 6a,b, it can be seen that the bottom of the joint resembles an ellipse, and there are two cavities connected by a protrusion in the center on the rotating punches side. Since the shape of the joint was not axisymmetric, in order to obtain the cross-sectional profile of the joint, it was cut from different positions using a wire cutting machine, i.e., from the A-A direction for the double-punch and the B-B direction for the single-punch shown in Figure 6a,b. The cross-sections obtained by cutting were then polished with sandpaper with different grain sizes, and the physical images of the cross-sectional profiles of the joint were taken with the PAIRSEN microscope with a magnification of 50–1000. As shown in Figure 6c,d, it can be seen that the A-A cross-sectional profile of the double-punch resembles the letter ‘π’, and the B-B profile of the single-punch resembles that of a traditional joint. The neck thickness (*T*_n_) and interlock value (*T*_u_) of the joints were measured by the measuring software with an accuracy of 0.01 mm.

### 3.4. Failure

**Figure 7:**
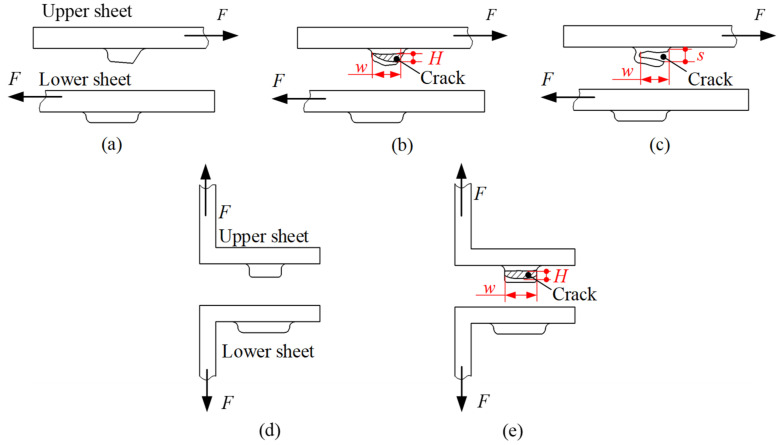
Schematic diagram of the failure forms of the upper sheet in shear and peel tests. (**a**) Unbuttoning, (**b**) tearing, (**c**) shearing, (**d**) unbuttoning and (**e**) tearing.

When the RCP joint is subjected to a shear and peel load, the upper and lower sheets eventually separate, resulting in joint failure. Since the upper sheet has a large deformation, it is most likely to fail in both the shear and peel tests. The main failure modes are shown in Figure 7, and *w*, *s* and *H* represent the dimensions of the crack. Figure 7a–c show the failure modes of the upper sheet in the shear test, which mainly include unbuttoning, tearing, and shearing. Figure 7d,e show the failure modes in the peel test, which mainly involves unbuttoning and tearing. The failure modes of the upper sheet can be judged from the crack. When *w* = 0, *s* = 0 and *H* = 0, the failure mode is neck unbuttoning; when *w* > 0, *H* > 0 and s = 0, the failure mode is neck tearing; when *w* > 0 and *s* > 0, the failure mode is shearing. When the failure mode was neck tearing or shearing, cracking was observed with the PAIRSEN (Shenzhen Superior Electronic Co., LTD, Shenzhen, China) microscope with a magnification of 50–1000, and the dimensions of the crack (*w*, *H*, *s*) were measured with the measuring software (HiView, version 1.4, Shenzhen, China) with an accuracy of 0.01 mm.

## 4. Results and Discussion

The shear and peel loads may directly reflect the strength of the RCP joints, while these loads are influenced by the cross-sectional profile dimensions and failure modes of the joints. The objective of this section is to evaluate the strengths of the RCP joints for each parameter from the three following aspects: shear and peel loads, cross-sectional profile dimensions, and failure modes.

### 4.1. Evaluation by Shear and Peel Loads

**Figure 8:**
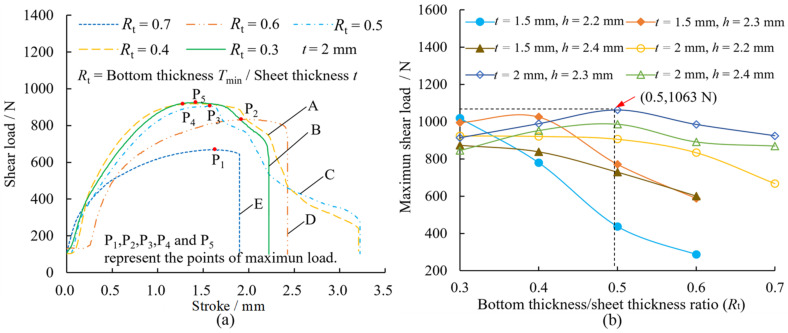
Variation of the shear loads on joints with (**a**) die depth *h* = 2.2 mm and (**b**) the bottom thickness/sheet thickness ratio (*R*_t_).

Figure 8a shows the shear load–stroke curves of the joints with a sheet thickness *t* = 2 mm and die depth *h* = 2.2 mm. The curves for the other parameters are similar and are not shown in this paper. The maximum shear load of each curve (points of P1–P5 in Figure 8) was considered as the shear strength of each respective joint. The maximum shear loads of joints with different parameters are shown in Figure 8b. The bottom thickness/sheet thickness ratio (*R*_t_) is 0.3, 0.4, 0.5, 0.6 and 0.7, respectively. When the sheet thickness was *t* = 1.5 mm, the joint failed to form in the case of *R*_t_ = 0.7, and there was no interlock between the upper and lower sheet. From Figure 8a, it can be seen that the shear load on the joint increases gradually with the stroke. After the maximum load is reached, there are two variation trends, one is a rapid drop after a certain fluctuation (curves A and C in Figure 8), and the other is a rapid drop (curves B, D and E in Figure 8). This is due to the differential deformation at the joint during shear (see Section 4.3 for details). High shear load means a high shear strength and high shear resistance of the joint. From Figure 8b, it can be seen that the shear load significantly increases with decreasing *R*_t_ in the case of *t* = 1.5 mm; while the shear load first increases and then decreases with decreasing *R*_t_ in the case of *t* = 2 mm. The overall shear loads of the joints are better in the case where *h* = 2.3 mm, and reach a maximum of 1063 N at *R*_t_ = 0.5 and *t* = 2 mm (red arrow in Figure 8), which corresponds to 39.4% of the shear load of the Al1060 sheet. The overall shear loads of the joints of sheet thickness *t* = 2 mm are better than when *t* = 1.5 mm.

**Figure 9:**
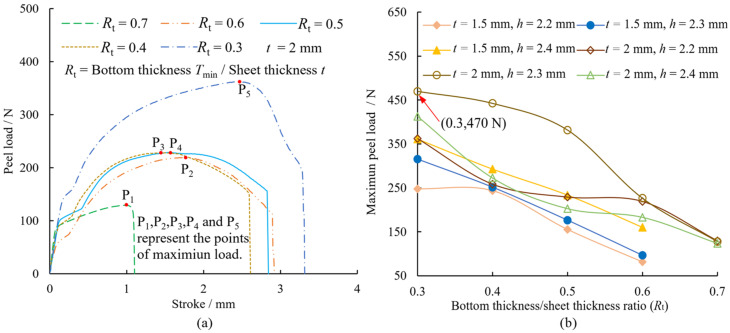
Variation of the peel loads on joints with (**a**) die depth *h* = 2.2 mm, *t* = 2 mm and (**b**) the bottom thickness/sheet thickness ratio.

Figure 9a shows the peel load-stroke curves of the joints for *t* = 2 mm and *h* = 2.2 mm. The curves of the other parameters were similar and are not shown in this paper. The maximum load on each curve (points of P1–P5 in Figure 9a) was considered as the peel strength of the joint. The maximum peel loads on joints with different parameters are shown in Figure 9b. When the sheet thickness *t* = 1.5 mm, the joint fails to form in the case of *R*_t_ = 0.7, and there is no interlock between the upper sheet and the lower sheet. From Figure 9a, it can be seen that the peel load on the joint increased gradually with the stroke, after which it reached the maximum and then quickly decreased to zero. From Figure 9b, it can be seen that in the case of *t* = 1.5 mm, the peel load increased approximately linearly with the decrease in *R*_t_. When *t* = 2 mm, the peel load on the joint is optimal and reaches a maximum of 470 N at *R*_t_ = 0.3 and *h* = 2.3 mm (red arrow in Figure 9) which corresponds to 17.5% of the peel load on the Al1060 sheet.

Comparing Figure 8 and Figure 9, it can be seen that the shear strength of the joint is higher and is approximately 2–7 times the peel strength. When the die depth *h* = 2.3 mm, the strength of the joint with *t* = 2 mm is superior to when *t* = 1.5 mm, and the shear strength and peel strength are best at *R*_t_ = 0.5 and 0.3, respectively. In the shear test, when the shear load is the dominant load, it is mainly concentrated on the upper sheet neck which has a similar shear effect; the base material itself plays a major role, resulting in a higher overall stiffness of the joint. However, in the peel test, where the peel load is the predominant load, it is mainly concentrated on the interlock of the joint. The friction between the two sheets plays a major role, less than that of the performance of the base metal itself, resulting in a lower overall stiffness of the joint. Table 1 and Table 2 show the maximum strokes and strokes at maximum load in the shear and peel tests. From the tables, it can be seen that in the shear and peel tests, the maximum stroke, and the stroke at maximum load, of the joints with *t* = 2 mm are generally smaller than when *t* = 1.5 mm. For the parameters, the maximum stroke, and the stroke at maximum load of the joints in the shear tests, are generally smaller than those of the joints in the peel tests, so that the joints have high strength and stiffness.

### 4.2. Evaluation by Cross-Sectional Profile Dimension

**Figure 10:**
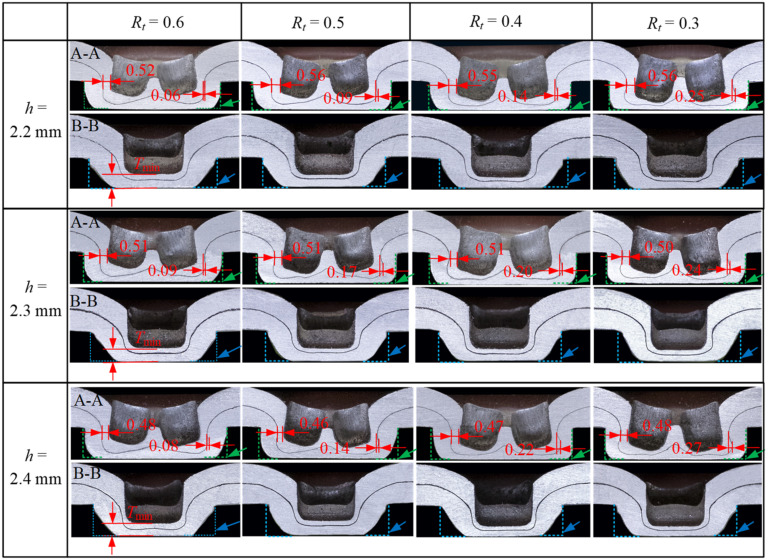
Cross-sectional geometries of joints of sheet thickness *t* = 1.5 mm at three die depths *h* and the four values of *R*_t_.

**Figure 11:**
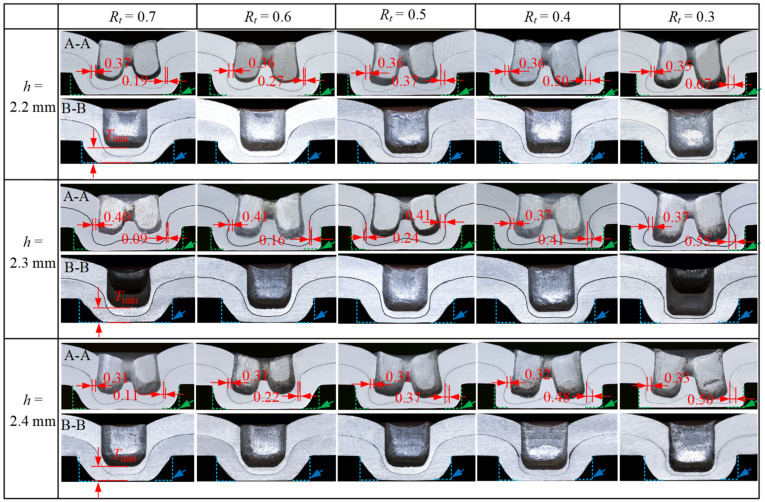
Cross-sectional geometries of joints of sheet thickness *t* = 2 mm at three die depths *h* and five values of *R*_t_.

**Figure 12:**
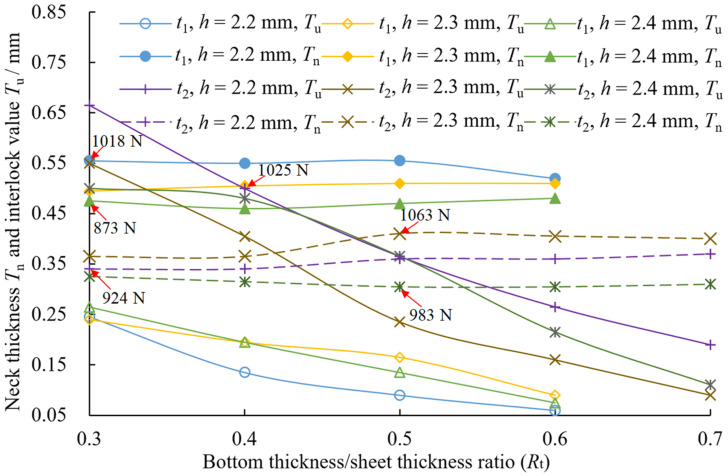
Variation of Interlock value and neck thickness of joints with *R*_t_.

The joints with a good strength should have adequate interlock, neck, and bottom thicknesses [22]. Figure 10 and Figure 11 show the cross-sectional profile of the joints with two sheet thicknesses at three die depths and five bottom thickness/sheet thickness ratios, and the definition of A-A and B-B is shown in Figure 6. When *t* = 1.5 mm, the joint failed to form in the case of *R*_t_ = 0.7, which is not shown in Figure 10. Figure 12 shows the neck thickness and interlock value variation with the *R*_t_ of the joints.

The interlock value varies with different parameters. From Figure 9 and Figure 10, it can be seen that for the same die depth, the interlock value of the cross-sectional profile A-A increased significantly as *R*_t_ decreased (i.e., the rotation angle increases). However, the interlock value on the cross-sectional profile B-B was insignificant (it is not measured in this study). This is because the forces generated by the rotating punches are mainly the resultant force in the direction of the double-punch and the die depth, which promotes the flow of material in these directions (the green arrow in Figure 10), favoring the formation of the interlock. In contrast, the component force in the direction of the single-punch is very small, so the material cannot flow as easily (the blue arrow in Figure 10), which is not conducive to the formation of the interlock. A small *R*_t_ value results in a large interlock value and thus a high peeling load (as shown in Figure 9b). When *R*_t_ < 0.5, the interlock value of the joint of sheet thickness *t* = 2 mm is approximately twice that of the joint of sheet thickness *t* = 1.5 mm on the cross-sectional profile A-A.

The neck thickness changes with different parameters. From Figure 11 and Figure 12, it can be seen that at the same die depth, the neck thickness does not change significantly when *R*_t_ decreases, and the average error of change is 1–6%. This is because the neck materials that flow in the vertical direction are blocked when the sheet material contacts the die bottom with decrease of *R*_t_, and the tensile stress is reduced, therefore the change in neck thickness is small. The neck thickness of the joint with *t* = 1.5 mm is greater than when *t* = 2 mm, and both reach their maximum at *h* = 2.2 mm and *h* = 2.3 mm, respectively. The neck thickness decreases slightly with increasing die depth at the same *R*_t_, since a deep die depth produces a large tensile stress on the neck material. The red arrows in Figure 12 represent the maximum shear loads of the joints at different parameters. From Figure 12, the maximum shear load occurs at the larger neck thickness. Comparing Figure 8 and Figure 12, it can be seen that for the same die depth, when the neck thickness is larger than the interlock value, the shear load increases with an increasing interlock value, while the shear load decreases slightly when the neck thickness is smaller than the interlock value.

In summary, the interlock value of the joint increases significantly with the decrease in *R*_t_, while the change of the neck thickness is small. The neck thickness of the joint of thin sheets (*t* = 1.5 mm) is greater than for thick sheets (*t* = 2 mm), while the interlock value is the opposite. A large interlock value results in a high peel load. The shear load is affected by both the neck thickness and the interlock value. When the neck thickness is larger than the interlock value, the shear load increases with an increasing interlock value. However, the shear load decreases slightly with an increasing interlock value.

### 4.3. Evaluation by Failure Mode

To obtain failure modes with a better shear and peel strength, it is necessary to analyze the types and characteristics of the RCP joint failure modes in shear and peel tests, as well as the relationship between failure modes, loads, and cross-sectional profiles.

**Figure 13:**
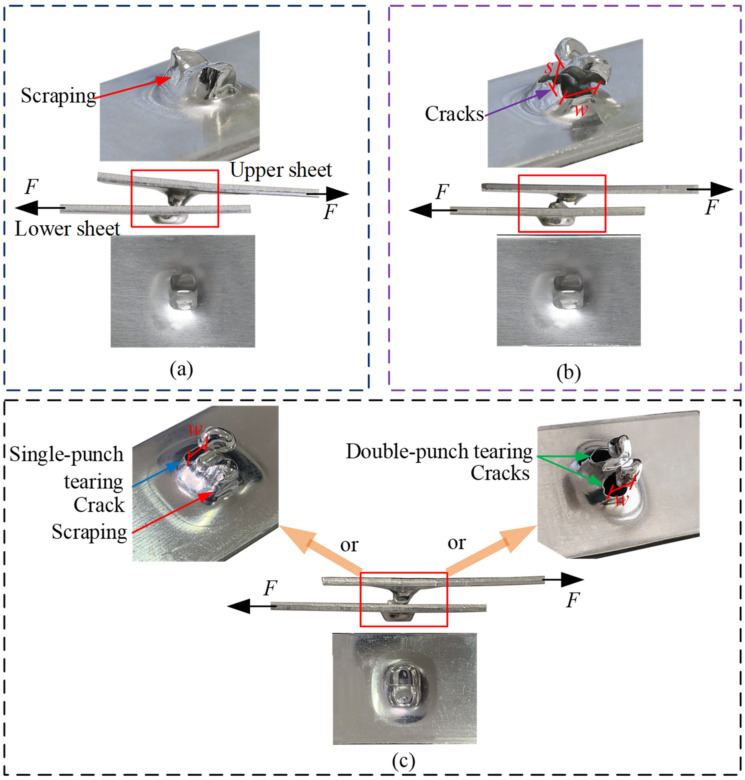
Failure modes of the joint in the shear tests. (**a**) Unbuttoning, (**b**) shearing and (**c**) tearing.

**Figure 14:**
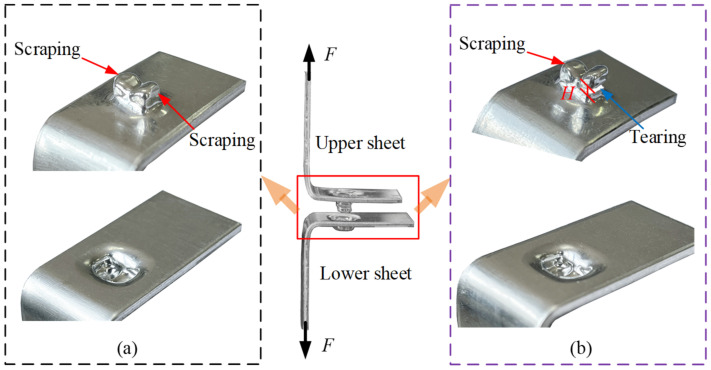
Failure modes of the joint in the peel tests. (**a**) Unbuttoning and (**b**) Single-punch tearing.

The typical failure modes of RCP joints in shear and peel tests are displayed in Figure 13 and Figure 14, respectively, which are mainly unbuttoning, tearing, and shearing. In Figure 13 and Figure 14, *s*, *w*, and *H* represent the dimensions of the crack when the joint fails. The peeling failure is shown in Figure 13a and Figure 14a. The upper sheet is only slightly scraped by the lower sheet (as shown by the red arrow in Figure 13a), and slight plastic deformation occurs without fracture in the neck. In the case of tearing failure, single-punch tearing and double-punch tearing occurs, as shown in Figure 13c and Figure 14b. The upper sheet is heavily scraped by the lower sheet, and severe plastic deformation occurs in the neck area. The single-punch (blue arrow) or double-punch (green arrow) in Figure 13c is torn (*w* > 0, *H* > 0), but the protrusion between the two punches has not been severed (*s* = 0). Shearing failure is shown in Figure 13b, which is different from tearing failure, and the protrusion between the two punches was also severed (*s* > 0). The characteristics of the different failure modes show that the failure of the joint depends mainly on that of the upper sheet (see Figure 7).

The failure modes are affected by the shear and peel loads of the joints, as shown in Table 3. The failure rates due to unbuttoning and tearing caused by single-punch tearing of the joints are 85.2% and 14.8%, respectively, in the peel test, and the unbuttoning, tearing, and shearing failure of the joints are 40.8%, 33.3% and 25.9%, respectively, in the shear test. In conjunction with the load analysis results of the joints in Section 4.1, it is found that the peel and shear load are small for unbuttoning failure, but large for tearing and shearing failure. Furthermore, the crack dimension increases with the load at the same die depth, as shown in Table 4. It is worth noting that only the failure modes with the highest probability were selected [23].

The failure mode of the joint is also affected by the cross-sectional profile dimensions. When the ratio of the neck thickness to the interlock value >2, the joint tends to be unbuttoned. When the ratio ~1, the joint tends to tear, while when the ratio ~0.6, the joint tends to fail mainly from shearing failure. The joint of the thick sheet fails mainly from tearing and shearing failure resulting in higher strength.

## 5. Conclusions

In this study, the shear strength and peel strength of Al1060 joints produced by RCP were evaluated. The main research results are summarized below:

(1) The joints with sheet thickness of 1.5 mm and 2 mm obtained higher shear strengths at *R*_t_ = 0.3–0.4 and *R*_t_ = 0.3–0.6, respectively. The joints with a sheet thickness of 2 mm exhibited better shear strengths, reaching the maximum of 1063 N at *h* = 2.3 mm and *R*_t_ = 0.5. The peel strength increases with decreasing *R*_t_, and when *R*_t_ = 0.3–0.5, the peel strength is higher. When *h* = 2.3 mm and *R*_t_ = 0.3, the peel strength of the joint with a sheet thickness of 2 mm reaches a maximum 470 N. The shear strengths are approximately 2–7 times the peel strength.
(2) With decreasing *R*_t_, the interlock value on the cross-sectional profile in the double-punch A-A direction increased significantly, while the interlock value on the cross-sectional profile in the single-punch B-B direction increased by only a little, and the neck thickness changes within a 6% error. The neck thickness of the joint of the thick sheet (*t* = 2 mm) was less than for the thin sheet (*t* = 1.5 mm), and the interlock value was reversed. The peel load is directly proportional to the interlock value, and the shear load is affected by both the neck thickness and the interlock value.
(3) There are three failure modes: unbuttoning, tearing, and shearing. In the peel tests, mainly unbuttoning occurs, while in the shear tests, mainly tearing and shearing failures were observed. When the ratio of the neck thickness to interlock value was ~0.6, the joints tend to fail by shearing. When the ratio is ~1, the joints were mainly torn. When the ratio >2, the joints tended to be unbuttoned. In the case of tear and shear failure, the joint strengths were higher.

The above results show that the mechanical load is a quantitative and reliable evaluation criterion, while the cross-sectional profile and failure mode are qualitative. The shear strength and peel strength of RCP joints are not ideal, and are affected by the shape of the joint. To improve the strength of the joint, the parameters of the rotating punch and die can be optimized; on the other hand, the process can be improved, such as by reducing the protrusion that connects the two cavities at the center of the joint to increase the interlock in the B-B direction cross-section.

## Figures and Tables

**Table 1:** Maximum strokes and strokes at maximum load in shear tests (units in mm).

*R*_t_	*h* = 2.2 mm		*h* = 2.3 mm		*h* = 2.4 mm	
	Max. Stroke *t* = 1.5/*t* = 2	Stroke at Max. Load *t* = 1.5/*t* = 2	Max. Stroke *t* = 1.5/*t* = 2	Stroke at Max. Load *t* = 1.5/*t* = 2	Max. Stroke *t* = 1.5/*t* = 2	Stroke at Max. Load *t* = 1.5/*t* = 2
0.3	3.22/2.39	1.46/1.38	3.83/2.61	2.63/1.84	2.5/1.72	1.40/0.74
0.4	1.87/3.21	1.43/1.38	3.87/2.42	2.14/2.22	2.43/2.67	1.78/1.04
0.5	1.21/3.22	1.07/1.55	2.61/3.35	1.54/1.90	1.97/2.67	1.42/1.48
0.6	0.41/2.43	0.19/1.96	1.56/3.69	1.78/2.81	1.03/3.50	1.18/1.85
0.7	―/1.9	―/1.62	―/3.48	―/2.14	―/3.07	―/2.18

**Table 2:** Maximum strokes and strokes at maximum load in peel tests (units in mm).

*R*_t_	*h* = 2.2 mm		*h* = 2.3 mm		*h* = 2.4 mm	
	Max. Stroke *t* = 1.5/*t* = 2	Stroke at Max. Load *t* = 1.5/*t* = 2	Max. Stroke *t* = 1.5/*t* = 2	Stroke at Max. Load *t* = 1.5/*t* = 2	Max. Stroke *t* = 1.5/*t* = 2	Stroke at Max. Load *t* = 1.5/*t* = 2
0.3	3.76/3.32	2.96/2.44	4.14/8.65	3.53/6.73	8.24/4.54	7.20/3.29
0.4	3.60/2.61	2.71/1.46	3.89/7.29	3.08/5.77	4.65/3.79	3.29/2.37
0.5	2.85/2.84	2.05/1.54	2.78/7.24	2.13/5.44	4.08/3.49	3.46/1.81
0.6	2.19/2.92	1.23/1.74	1.92/6.60	1.10/4.9	2.49/3.15	1.95/1.58
0.7	―/1.1	―/0.98	―/2.87	―/1.79	―/2.69	―/1.36

Note: “―” indicates that the joint failed to form, *R*_t_—ratio between bottom thickness and sheet thickness, *h*—die depth, *t*—sheet thickness.

**Table 3:** Number of joint failures by failure type in shear and peel tests.

Sheet Thickness /mm	Die Depth /mm	Sample Number	Peel Test/Shear Test			
			Unbuttoning	Single-Punch Tearing	Double-Punch Tearing	Shearing
1.5	2.2	12	12/12	NA/NA	NA/NA	NA/NA
	2.3	12	12/9	NA/3	NA/NA	NA/NA
	2.4	12	9/9	3/3	NA/NA	NA/NA
2	2.2	15	15/3	NA/3	NA/9	NA/NA
	2.3	15	12/NA	3/3	NA/NA	NA/12
	2.4	15	9/NA	6/6	NA/NA	NA/9

**Table 4:** Shear loads, crack dimensions, and joint failure types of sheet thickness *t* = 2 mm in shear tests.

*R*_t_	*h* = 2.2 mm/*h* = 2.3 mm/*h* = 2.4 mm		
	Shear Loads/N	Crack Dimensions/mm	Failure Types
0.3	924/913/845	3.2/2.40/3.32	□/▽/▽
0.4	921/968/952	2.9/3.58/3.54	□/▽/▽
0.5	906/1063/986	1.9/4.72/3.58	□/▽/▽
0.6	834/989/891	1.7/3.62/3.52	▲/▽/▲
0.7	668/924/859	0/2.80/2.48	○/▲/▲

Note: ○—Unbuttoning, ▽—Shearing, □—Double-punch tearing, ▲—Single-punch tearing; *R*_t_—bottom thickness/sheet thickness, *h*—die depth.

## Data Availability

Not applicable.

## References

[1] Moradi M., Meiabadi M.S., Demers V. (2021). A numerical investigation of friction stir welding parameters in joining dissimilar aluminium alloys using finite element method. Int. J. Manuf. Res..

[2] Vahdati M., Moradi M., Shamsborhan M. (2020). Modeling and Optimization of the Yield Strength and Tensile Strength of Al7075 Butt Joint Produced by FSW and SFSW Using RSM and Desirability Function Method. Trans. Indian Inst. Met..

[3] He Y.L., Yang L.F., Zong P.J., Dang J., Ma J.P. (2022). Rotated clinching process for two-layer metallic sheets. Int. J. Adv. Manuf. Technol..

[4] Carboni M., Beretta S., Monno M. (2006). Fatigue behaviour of tensile-shear loaded clinched joints. Eng. Fract. Mech..

[5] Abe Y., Saito T., Mori K.I., Kato T. (2018). Mechanical clinching with dies for control of metal flow of ultra-high-strength steel and high-strength steel sheets. Proc. Inst. Mech. Eng. Part B J. Eng. Manuf..

[6] Mori K., Abe Y., Kato T. (2012). Mechanism of superiority of fatigue strength for aluminum alloy sheets joined by mechanical clinching and self-pierce riveting. J. Mater. Process. Technol..

[7] Su Z.M., Lin P.C., Lai W.J., Pan J. (2015). Fatigue analyses of self-piercing rivets and clinch joints in lap-shear specimens of aluminum sheets. Int. J. Fatigue.

[8] Lambiase F. (2013). Influence of process parameters in mechanical clinching with extensible dies. Int. J. Adv. Manuf. Technol..

[9] Wen T., Wang H., Yang C., Liu L.T. (2014). On a reshaping method of clinched joints to reduce the protrusion height. Int. J. Adv. Manuf. Technol..

[10] Zhao S., Xu F., Tohru I., Chao C., Zhao X., Han X. (2017). Comparative study on two compressing methods of clinched joints with dissimilar aluminum alloy sheets. Int. J. Adv. Manuf. Technol..

[11] Li Q., Xu C., Gao S., Han X., Ma F., Gu D., Zhao Q. (2022). Research on the forming quality of clinched joint for dissimilar sheet metal. Int. J. Adv. Manuf. Technol..

[12] Jiang T., Liu Z.X., Wang P.C. (2015). Quality inspection of clinched joints of steel and aluminum. Int. J. Adv. Manuf. Technol..

[13] He X.C., Zhao L., Yang H.Y., Xing B.Y., Wang Y.Q., Deng C.J., Gu F.S., Ball A. (2014). Investigations of strength and energy absorption of clinched joints. Comp. Mater. Sci..

[14] Ran X., Chen C., Zhang H., Ouyang Y. (2021). Investigation of the clinching process with rectangle punch. Thin Wall Struct..

[15] Peng H., Chen C., Li H., Gao X. (2021). Joining thin-walled structures without protuberance by two-strokes flattening clinching process. Int. J. Adv. Manuf. Technol..

[16] Lee C.J., Lee J.M., Lee S.H., Kim B.H., Kim B.M., Ko D.C. (2014). Design of hole-clinching process for joining CFRP and aluminum alloy sheet. Int. J. Precis. Eng. Man..

[17] Liu Y., Zhuang W.M., Wu S.J. (2020). Effects of hole diameter and ply angle on the mechanical behaviour of hole-clinched joints in carbon fibre reinforced polymers and aluminum alloy sheets. Int. J. Adv. Manuf. Technol..

[18] Chen C., Zhang H.Y., Qin D.L. (2021). Joining different aluminum alloy sheets by flat clinching process. Int. J. Adv. Manuf. Technol..

[19] Chen C., Ouyang Y.W., Qin D.L. (2021). Finite element analysis of material flow in flat-rivet clinching process. Int. J. Adv. Manuf. Technol..

[20] Babalo V., Fazli A., Soltanpour M. (2021). Experimental study of the mechanical performance of the new highspeed mechanical clinching. Int. J. Lightweight Mater. Manuf..

[21] Zhao L., He X.C., Lu Y. (2014). Research of mechanical behavior for rounded and rectangular clinched joint. Adv. Mat. Res..

[22] Aris J.V. (2006). Ensuring the integrity in clinching process. J. Mater. Process. Technol..

[23] Carboni M., Annoni M. (2013). Ultrasonic metal welding of AA6022-T4 lap joints: Part II—Fatigue behaviour, failure analysis and modelling. Sci. Technol. Weld. Join..

